# Combined signature of N7-methylguanosine regulators with their related genes and the tumor microenvironment: a prognostic and therapeutic biomarker for breast cancer

**DOI:** 10.3389/fimmu.2023.1260195

**Published:** 2023-10-05

**Authors:** Tingjun Li, Zhishan Chen, Zhitang Wang, Jingyu Lu, Debo Chen

**Affiliations:** ^1^ The School of Clinical Medicine, Fujian Medical University, Fuzhou, China; ^2^ Department of Breast Surgery, Quanzhou First Hospital of Fujian Medical University, Quanzhou, China; ^3^ Department of Breast and Thyroid Surgery, Nan’an Hospital, Quanzhou, China; ^4^ Department of Breast Surgery, The Affiliated Hospital of Putian University, Putian, China

**Keywords:** breast cancer, N 7 -methylguanosine modification, tumor microenvironment, prognosis, immunotherapy

## Abstract

**Background:**

Identifying predictive markers for breast cancer (BC) prognosis and immunotherapeutic responses remains challenging. Recent findings indicate that N^7^-methylguanosine (m7G) modification and the tumor microenvironment (TME) are critical for BC tumorigenesis and metastasis, suggesting that integrating m7G modifications and TME cell characteristics could improve the predictive accuracy for prognosis and immunotherapeutic responses.

**Methods:**

We utilized bulk RNA-sequencing data from The Cancer Genome Atlas Breast Cancer Cohort and the GSE42568 and GSE146558 datasets to identify BC-specific m7G-modification regulators and associated genes. We used multiple m7G databases and RNA interference to validate the relationships between BC-specific m7G-modification regulators (METTL1 and WDR4) and related genes. Single-cell RNA-sequencing data from GSE176078 confirmed the association between m7G modifications and TME cells. We constructed an m7G-TME classifier, validated the results using an independent BC cohort (GSE20685; *n* = 327), investigated the clinical significance of BC-specific m7G-modifying regulators by reverse transcription-quantitative polymerase chain reaction (RT-qPCR) analysis, and performed tissue-microarray assays on 192 BC samples.

**Results:**

Immunohistochemistry and RT-qPCR results indicated that METTL1 and WDR4 overexpression in BC correlated with poor patient prognosis. Moreover, single-cell analysis revealed relationships between m7G modification and TME cells, indicating their potential as indicators of BC prognosis and treatment responses. The m7G-TME classifier enabled patient subgrouping and revealed significantly better survival and treatment responses in the m7G^low^+TME^high^ group. Significant differences in tumor biological functions and immunophenotypes occurred among the different subgroups.

**Conclusions:**

The m7G-TME classifier offers a promising tool for predicting prognosis and immunotherapeutic responses in BC, which could support personalized therapeutic strategies.

## 1 Introduction

Breast cancer (BC) is the most common cancer in women worldwide with an incidence rate that has continued to increase steadily (1). In 2020, an estimated 2.3 million new BC cases were diagnosed globally, accounting for 11.7% of all cancer diagnoses. By 2040, this number is expected to exceed 3 million (2). BC treatment has progressed from a single surgical approach to multifaceted strategies involving local and systemic therapies. Unlike traditional chemotherapy, immune checkpoint inhibitors (ICIs) have shown potential as adjunctive therapies, with minimal toxicity or adverse effects. Although recent data have suggested that patients with BC may benefit from immunotherapy (3), predictive markers for guiding treatment selection are lacking. Hence, developing novel prognostic biomarkers and diagnostic tools may revolutionize BC management.

With the recent development of bioinformatics technology, an increasing number of datasets on RNA modification have emerged, and their role in disease development has become clearer (4–8). One of the most prevalent RNA modifications is N^7^-methylguanosine (m7G), essential for RNA processing, degradation, and translation in eukaryotes (9). Methyltransferase-like 1 (METTL1) interacts with WD repeat structural domain 4 (WDR4) to add m7G modifications to the internal regions of several types of RNA. RNA guanine-7 methyltransferase (RNMT) and RNA guanine-7 methyltransferase activation subunit (RAMAC) are primarily responsible for adding m7G to the mRNA 5′ cap. Williams–Bern syndrome chromosome region 22 (WBSCR22, also known as BUD23) and tRNA methyltransferase activation unit 11-2 (TRMT112) are involved in the m7G modification of rRNA (10). Recently, quaking proteins (QKIs) were reported to act as m7G readers by selectively recognizing m7G modifications within mRNA and regulating target mRNA metabolism and cellular drug resistance (11).

m7G modifications have been linked to tumor progression and development. In BC, increased RNMT activity is significantly associated with the oncogenic mutation rate of *PIK3CA*, suggesting that RNMT-targeted therapy may have better developmental prospects for patients with *PIK3CA* mutations (12). Similarly, *METTL1* expression is significantly upregulated in BC (13). The impact of the expression signature of various m7G regulators and m7G-associated non-coding RNAs on BC prognosis has also been reported (14–17). Furthermore, METTL1 specifically modulates the translation of oncogenic transcripts associated with epidermal growth factor receptor pathways and cell-cycle progression by mediating m7G tRNA modifications in intrahepatic cholangiocarcinoma (18). In hepatocellular carcinoma, WDR4 facilitates the binding of eukaryotic translation initiation factor 2A to cyclin B1 (*CCNB1*) mRNA, promoting its transcription and enhancing diverse malignant phenotypes (19).

Intricate interactions between cancer cells and the tumor microenvironment (TME) underlie the multifactorial processes of tumorigenesis and progression (20). The TME and immune regulation are crucial factors that affect BC immunotherapy (21). Among the different types of immune cells, tumor-infiltrating lymphocytes (TILs) are promising predictive and prognostic markers for various BC subtypes. In HER2-positive and triple-negative BCs, higher TIL levels are significantly associated with improved overall survival (OS), fewer recurrences, and higher rates of pathological complete remission after neoadjuvant therapy (22–24). In addition to lymphocytes, tumors harbor many myeloid cells, including dendritic cells and macrophages. In particular, tumor-associated macrophages (TAMs) can negatively affect the disease-free survival (DFS) and OS of patients with BC (25, 26).

The m7G modification is closely associated with immune cell infiltration into the TME. Recently, METTL1 was found to be upregulated in radioresistant hepatocellular carcinoma and to recruit bone marrow-derived suppressor cells by enhancing transforming growth factor (TGF)-β2 translation, creating an immunosuppressive environment (27). Nevertheless, to date, no studies have investigated the TME in BC through a combined analysis of m7G modifications and the cellular landscape. In this study, we investigated the association between m7G regulators with their associated genes and TME cells and predicted the prognosis and treatment response of BC by combining their signatures. Our findings may advance our understanding of tumor-specific biology based on m7G modifications and the TME, with important implications for the clinical management of BC.

## 2 Materials and methods

### 2.1 Data sources

We obtained data from the Gene Expression Omnibus (GEO) (https://www.ncbi.nlm.nih.gov/geo/) and The Cancer Genome Atlas (TCGA) (https://portal.gdc.cancer.gov/) databases. Using TCGA Breast Cancer (TCGA-BRCA) database, we conducted a differential analysis of RNA-sequencing (RNA-seq) data from 113 adjacent normal tissue samples and 1113 BC samples. Co-expression analyses were performed using BC samples from TCGA-BRCA (*n* = 1113) and two BC microarray datasets (GSE42568 [*n* = 104] and GSE146558 [*n* = 106]). We also obtained three samples totaling 14,281 cells (sample ident: CID44971, CID4513, and CID4523) from the BC single-cell dataset GSE176078 with cell annotations (28) for single-cell analysis through random sampling. For subsequent analyses, patients with survival information in TCGA-BRCA cohort (*n* = 1043) were used as the training cohort, and an independent dataset (GSE20685) containing information for 327 BC samples was used to validate the classifier model. Microarray data were log-transformed, and RNA sequencing data were collected to obtain transcripts-per-million values. We studied samples from 192 patients with BC (treated at Quanzhou First Hospital of Fujian Medical University between October 2012 and November 2021) for our tissue microarray (TMA) analysis. Patient clinical information, including the histological tumor grade, clinical stage (as defined by the American Joint Committee on Cancer, 8th edition), and follow-up recurrence and survival data, were obtained from electronic medical records. Patient information is presented in detail in **Table 1**.

**Table 1:** Patient characteristics of tissue microarray.

Characteristic	METTL1 (*n* = 96)	WDR4 (*n* = 96)	*p* value
Age			
**Mean (SD)**	52.3 (9.87)	52.1 (11.0)	0.863
**Median [Min, Max]**	51.0 [29.0, 75.0]	51.0 [27.0, 84.0]	
Grade			
**I**	5 (5.2%)	5 (5.2%)	0.865
**II**	55 (57.3%)	56 (58.3%)	
**III**	23 (24.0%)	28 (29.2%)	
**Missing**	13 (13.5%)	7 (7.3%)	
Stage			
**0–I**	38 (39.6%)	32 (33.3%)	0.655
**II**	38 (39.6%)	43 (44.8%)	
**III–IV**	20 (20.8%)	21 (21.9%)	
State			
**No recurrence**	86 (89.6%)	84 (87.5%)	0.999
**Recurrence**	10 (10.4%)	10 (10.4%)	
**Missing**	0 (0%)	2 (2.1%)	

This study adhered to the principles outlined in the Declaration of Helsinki and was approved by the Medical Ethics Committee of Quanzhou First Hospital of Fujian Medical University (No 2022-208). All patients provided written informed consent before participating in the study.

### 2.2 Identifying differentially expressed and prognosis-related genes and constructing a co-expression network

To identify genes that regulate m7G RNA methylation that were differentially expressed between tumor and normal tissues, we first identified m7G regulators (METTL1, WDR4, RNMT, RAMAC, WBSCR22, and TRMT112) in the literature (9). Differential analyses were performed using the Limma package of R software (version 4.1.1). Additionally, univariate Cox regression analysis was performed to identify m7G regulators associated with prognosis. We selected genes that met the following criteria as regulators of BC-specific m7G RNA methylation: *p<*0.05 and |log_2_ (fold-change in expression)| >0.5 (for differential expression analysis) and *p<* 0.05 (for univariate Cox regression analysis). Co-expression analyses were performed on the selected genes. We identified m7G regulator-related genes in the GSE42568, GSE146558, and TCGA-BRCA datasets using Pearson correlation analysis and retained those with a correlation coefficient (*r*) > 0.4 and *p<* 0.001. We designated the BC-specific m7G regulators and their co-expressed genes as m7G regulator-related genes (MGRRGs). Cytoscape (version 3.9.1) was used to visualize the co-expression relationships, whereas the clusterProfiler package (version 4.0.5) was utilized to analyze MGRRGs for enrichment in terms of Kyoto Encyclopedia of Genes and Genomes (KEGG) pathways and Gene Ontology biological processes (GO-BP).

### 2.3 Database examination for regulatory links between m7G regulators and MGRRGs

Evidence supporting the regulation of MGRRGs by m7G regulators was obtained from the m7GHub v2.0 (4), RMVar (29), RMDisease v2.0 (8), and RM2Target (30) databases. The m7GHub v2.0 database contains comprehensive data for the study of m7G modifications on internal mRNAs. The RMVar and RMDisease v2.0 databases provide data on many genomic variants that may affect RNA modifications, whereas the RM2Target database provides target-gene data for RNA-modifier regulators. In further analysis, only MGRRGs with confirmed m7G modifier loci in more than one database or with prior experimental validation of m7G-modifier regulation were considered.

### 2.4 Single-cell RNA-seq data analysis, m7G-score calculations, and cell–cell communication analysis

Three filters were applied to the raw matrix for each cell to ensure data quality in terms of unique molecular identifiers (from 100 to 100,000), gene counts (from 300 to 8,000), and mitochondrial genes (≤ 15%). Using the MGRRGs, we calculated the m7G scores for each cell subset using the AddModuleScore function of the Seurat package (version 4.1.0) (31). Tumor cells were categorized based on their m7G scores, with those > the 75th percentile assigned to the m7G-high group and those< the 25th percentile assigned to the m7G-low group. Gene-set enrichment analysis (GSEA) of different tumor cell subgroups was performed using the ‘h.all.v2022.1.Hs.symbols.gmt’ and ‘c5.go.bp.v2022.1.Hs.symbols.gmt’ gene sets from MSigDB and the fgsea package of R software. CellChat (version 1.1.3) (32) was used to infer differences in ligand–receptor interactions and signaling pathways across different subgroups of tumor cells and other cell subsets.

### 2.5 Identification of prognosis-related MGRRGs and TME cells

We downloaded an expression matrix for all cells from the single-cell RNA (scRNA)-seq data. To generate the CIBERSORTx signature matrix with TME cell types and utilized the Create Signature Matrix module at https://cibersortx.stanford.edu/runcibersortx.php (33). The generated signature matrix was then leveraged for CIBERSORTx-based deconvolution of TCGA-BRCA cohorts. The deconvolution scores calculated using CIBERSORTx were used to determine the abundance of each TME cell type in each sample. To select MGRRGs and TME cells associated with BC survival, we performed univariate Cox regression analysis using a significance level of *p<* 0.05.

### 2.6 Establishment of m7G scores, TME scores, and the m7G-TME classifier

We conducted multivariate Cox regression analysis on prognosis-related MGRRGs and TME cells to obtain the corresponding regression coefficients. The variance of the multivariate Cox regression coefficients was calculated after 1000 bootstrap samplings. The m7G and TME scores were based on previously reported data. The weight of each prognosis-related MGRRG in the m7G score was determined by its respective regression coefficient and the corresponding variance from 1000 bootstrap samplings. The m7G score was calculated as per Equation (1):


(1) $$\text{m}7\text{G score }=\sum_{i=1}^{n}\frac{Coefi*Gi}{Bootstrap\left(SD\right)},$$

Similarly, the TME score was calculated using Equation (2):


(2) $$\text{TME score }=\sum_{j=1}^{n}\frac{-\text{Coefj}*\text{Cj}}{Bootstrap\left(SD\right)},$$

where Gi and Cj represent the abundance of MGRRG gene i and TME cell j in each sample, respectively. SD represents standard deviation.

The m7G and TME scores were integrated to develop the m7G-TME classifier. We subsequently stratified the tumors into the following subgroups based on the median m7G and TME scores in each cohort: m7G^high^+TMEl^ow^, intermediate-mixed (m7G^high^+TME^high^ and m7G^low^+TME^low^), and m7G^low^+TME^high^.

### 2.7 Functional-enrichment analysis of the tumor cell molecular signatures

We performed gene-set variation analysis (GSVA) with the ‘h.all.v2022.1.Hs.symbols.gmt,’ ‘c2.cp.kegg.v2022.1.Hs.symbols.gmt,’ and ‘c5.go.bp.v2022.1.Hs.symbols.gmt’ gene sets from MSigDB to conduct our analysis. Using the GSVA package of R software, we performed GSVA enrichment analysis to evaluate the biological functions of different groups of tumor samples. Additionally, heatmaps were generated using the pheatmap package of R software for visualization purposes.

### 2.8 Analysis of tumor immunophenotypes and predicting ICI-treatment responses

We compared the expression levels of human leukocyte antigens (HLAs) (34) and immune checkpoints (35) in different subgroups of BC samples. Cancer–immunity-cycle analysis was conducted using TIP (Tracking Tumor Immunophenotype) (http://biocc.hrbmu.edu.cn/TIP/) (36), which conceptualizes anti-cancer immune response as a series of stepwise events referred to as the cancer–immunity cycle. Using the TIP website, we compared differences in the seven-step cancer–immunity cycle between the groups. We then used the TIDE (Tumor Immune Dysfunction and Exclusion) website (http://tide.dfci.harvard.edu/) (37) to predict the responses to ICI treatment with these samples. To compare gene-expression levels between subgroups, we used a web tool (https://bionic-vis.biologie.uni-greifswald.de/) (38) to generate proteomaps.

### 2.9 Statistical analysis

Statistical analyses were performed using R software (version 4.1.1), and the survival rate of each group was assessed using the log-rank test. Comparisons of more than two groups were conducted using the Kruskal–Wallis test, whereas comparisons of two groups were carried out using the Wilcoxon test. We generated survival curves for each subgroup in the dataset using the Kaplan–Meier method. To analyze the frequency of ICI-treatment responses between the m7G-TME classifier subgroups, we used the chi-squared test. The threshold for statistical significance was set at *p<* 0.05. The parameters used for the analysis described above are the default parameters.

### 2.10 Cell culture

We utilized the mammary gland epithelial cell line MCF-10A (RRID: CVCL_0598) and the BC cell line MCF-7 (RRID: CVCL_0031), procured from the Cell Resource Center of Shanghai Institutes for Biological Sciences. MCF-7 cells were cultured in Minimal Essential Media (MEM; SH30024.FS, Hyclone, USA) containing 10% fetal bovine serum (FBS; BS1615-119, BIOEXPLORER, USA), and MCF 10A cells were cultured in a specific epithelial culture medium (CL-0525, Procell Co., China). All cells were incubated at 37 °C with 5% CO_2_.

### 2.11 Quantitative reverse transcription-quantitative polymerase chain reaction analysis

RNA samples were isolated using an RNApure Tissue & Cell Kit (DNase I) (CW0560S, CWBIO, China). Reverse transcription was performed using a PrimeScript RT Reagent Kit (RR047A, Takara, Japan). Quantitative PCR was performed using the SYBR Green PCR Master Mix (RR820A, Takara, Japan) on a StepOnePlus System (Applied Biosystems). The 2^−ΔΔCT^ method was used to determine fold changes in gene expression levels, with normalization to glyceraldehyde-3-phosphate dehydrogenase (*GAPDH*) mRNA expression as an internal control. **Supplementary Table 1** provides the primer sequences used in this study. Each PCR was conducted in triplicate.

### 2.12 Immunohistochemical analysis of TMA

The TMAs were constructed by Shanghai Outdo Biotech Co., Ltd., Shanghai, China. Antibodies against METTL1 (diluted 1:5000, ab271063, Abcam, UK) and WDR4 (diluted 1:1000, ab169526, Abcam, UK) were used for IHC staining. Immunostaining was considered positive if ≥ 10% of the tumor cells were immunoreactive. Two investigators independently analyzed the IHC results using a double-blind method without knowledge of the clinical and pathological characteristics of the patients. The staining intensity and percentage of positive cells were used to evaluate METTL1 and WDR4 expression. The proportion of positive cells was classified and scored as 0–10% (0), 11–30% (1), 31–50% (2), 51–80% (3), and 81–100% (4). The staining intensity was rated as no staining (0), weak staining (1), moderate staining (2), or strong staining (3). The total score was calculated by multiplying the percentage of positively-stained cells by the staining intensity.

### 2.13 RNA interference and transfections

We acquired small-interfering RNAs (siRNAs) from RiboBio (Guangzhou, China) designed to target the mRNA sequences of METTL1 and WDR4. The riboFECT™ CP Transfection Kit (C10511-05 & C10502-05, RiboBio, Guangzhou, China) was used to transfect MCF-7 cells with siRNAs. We conducted qPCR at 72 h post-transfection. **Supplementary Table 2** presents the sequences of the siRNA used in this study.

## 3 Results

### 3.1 Identification of BC-specific m7G regulators and their co-expression network

**Figure 1:**
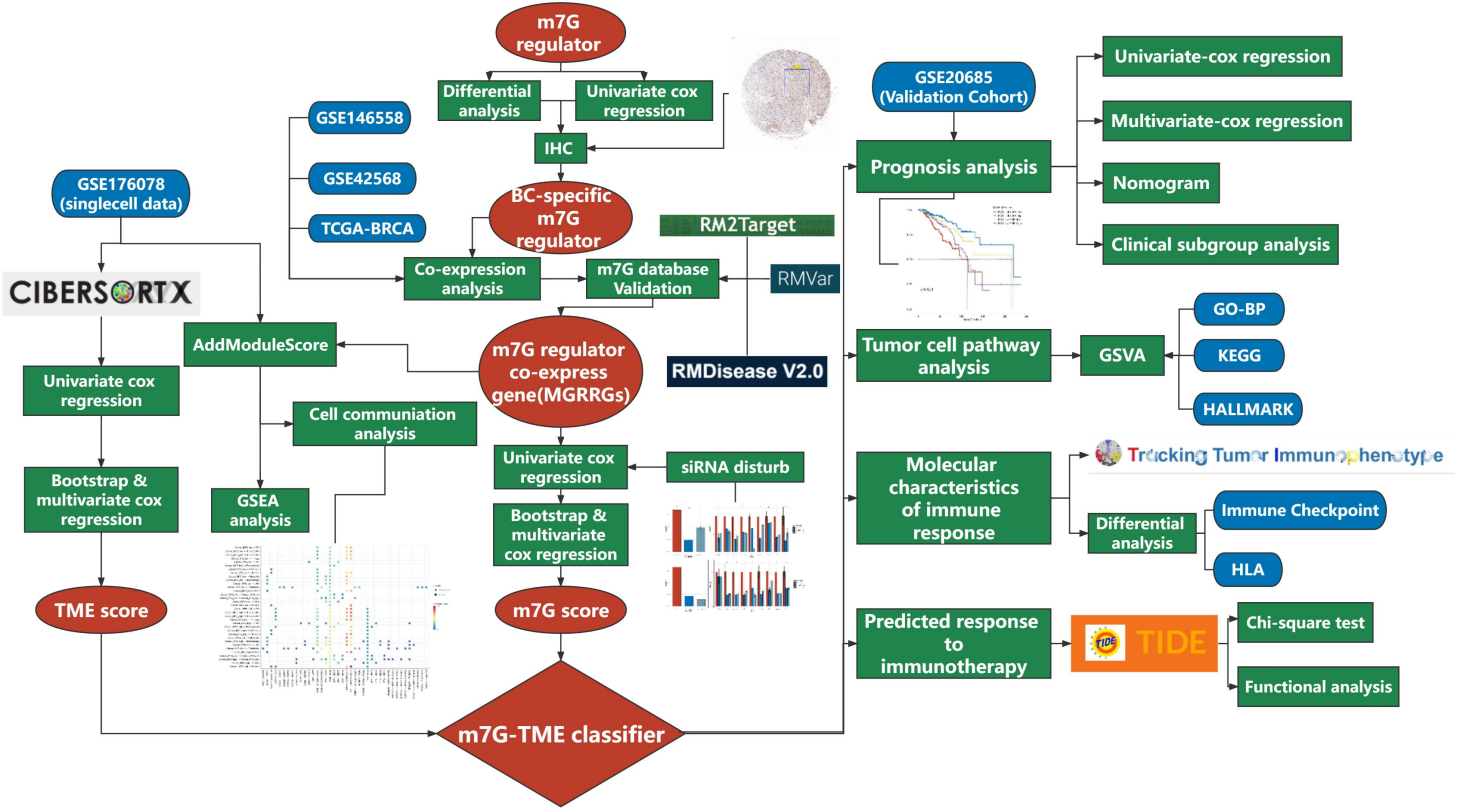
Flow chart of the present study.

**Figure 2:**
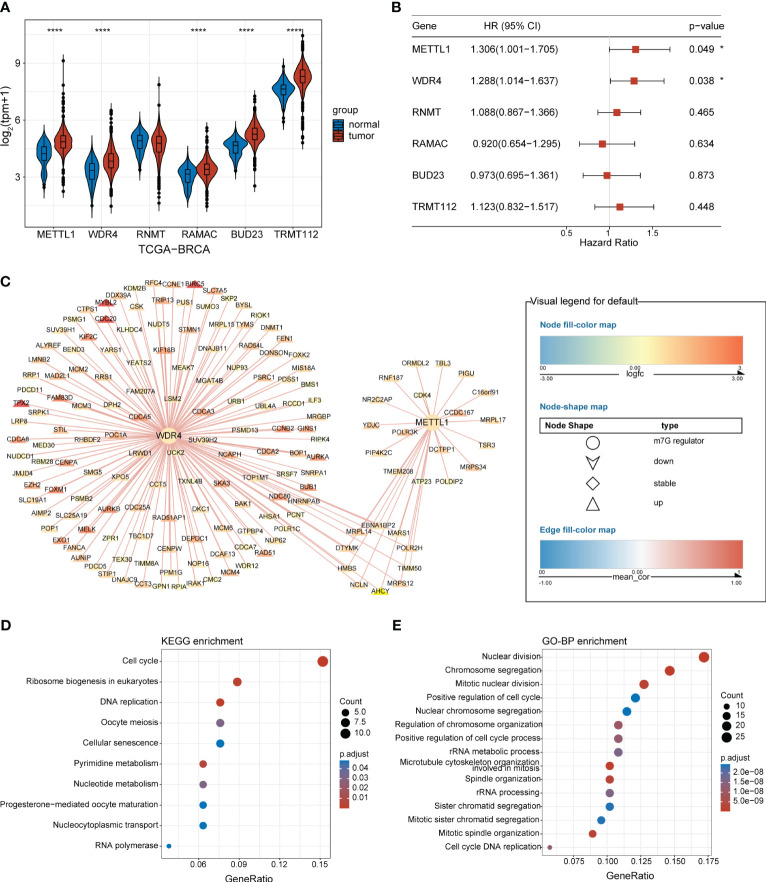
BC-specific m7G regulators and their co-expression network. **(A)** Violin plot showing the differential expression of m7G RNA-methylation regulators in BC and normal tissues. **(B)** Forest plot presenting univariate Cox regression analysis of the m7G-methylation regulators. **(C)** Network showing connections between BC-specific m7G regulators and MGRRGs. The node colors reflect the fold-differences in expression between BC and normal tissues, whereas the edge colors represent the correlation coefficients with m7G regulators. **(D)** KEGG and **(E)** GO-BP enrichment results. *p< 0.05; ****p< 0.0001.

An overview of the study design is presented in **Figure 1**. Six m7G regulators were identified from a literature search. We analyzed the expression levels of all six m7G regulators in normal and tumor tissues, as well as their association with patient prognosis. Our findings revealed that *METTL1* and *WDR4* were highly expressed in BC tissues (**Figure 2A**) and their high expression levels indicated a poor prognosis (**Figure 2B**). Co-expression analysis was performed on three public datasets, and the results were screened against the m7G database (**Supplementary Table 3**), revealing 165 MGRRGs (**Figure 2C**). All 165 co-expressed genes correlated positively with the m7G regulators, of which 126 were highly expressed in BC. KEGG analysis indicated that the MGRRGs were significantly enriched for pathways related to cell-cycle progression (**Figure 2D**). Furthermore, GO-BP enrichment analyses of the MGRRGs revealed their involvement in nuclear division, chromosome segregation, and positive regulation of the cell cycle (**Figure 2E**). Our results indicate that increased levels of MGRRGs may significantly affect cell-cycle progression in cancer cells, potentially contributing to the development of BC.

### 3.2 Differential expression of MGRRGs at the cellular level and implications for cellular communication

**Figure 3:**
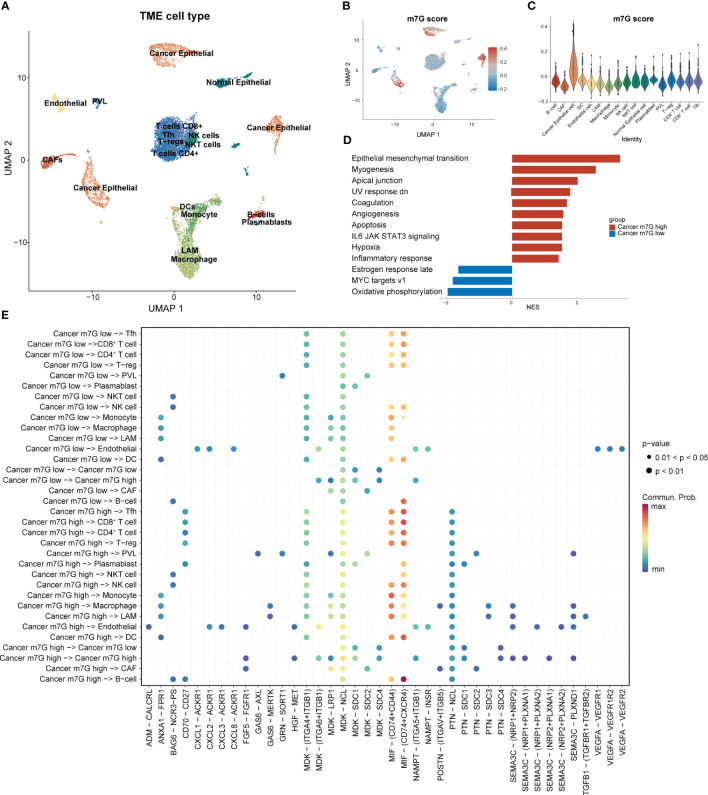
Differential expression of MGRRGs at the cellular level and implications for cellular communication. (PVL. perivascular-like cell; T-regs, regulatory T cell; Tfh, T follicular helper cell) **(A)** UMAP plot of all cells with annotation based on the original dataset (GSE176078). **(B)** UMAP plot of all cells with colors reflecting their m7G scores. **(C)** Violin plot showing m7G scores of cells from different cell types. Cancer epithelial cells had the highest scores. **(D)** GSEA results for the Hallmark gene set of cancer cells with different m7G scores. **(E)** Results of cell-communication analysis between tumor cells and non-tumor cells.

Following strict quality-control criteria, we screened the processed scRNA-seq data using the original metadata file to annotate all cells. After gene filtering, normalization, and principal-component analysis, we employed uniform manifold approximation and projection (UMAP) plots to demonstrate the cell clustering of the samples (**Figure 3A**). Additionally, UMAP and violin plots were used to show the overall expression levels of MGRRGs in different cell types, with elevated expression observed in tumor cells (**Figures 3B, C**). Tumor cells were grouped based on MGRRG-expression levels, and GSEA was used to assess functional differences between the different groups (**Figure 3D**; **Supplementary Figure 1**). Our findings revealed that epithelial–mesenchymal transition, angiogenesis, and Wnt pathways were significantly enriched in tumor cells with high m7G scores, whereas oxidative phosphorylation, negative regulation of gene expression, and positive regulation of intrinsic apoptotic pathways were enriched in tumor cells with low m7G scores. We further constructed a cell-communication network using receptor–ligand-pair interactions of secreted signals through CellChat to investigate the molecular associations between tumor cells and other cells (**Figure 3E**). Notably, tumor cells in the high-MGRRG-score group exhibited higher outgoing interaction strengths, with stronger probabilities of communicating through signaling pathways, such as the macrophage migration inhibitory factor (MIF), fibroblast growth factor 5 (FGF5), transforming growth factor-beta (TGF-β), and semaphorin 3C (SEMA3C) pathways, than cells in the low-MGRRG-score group. Overall, we hypothesized that tumor cells in the high-MGRRG-score group would have increased proliferative and invasive abilities, while possessing stronger abilities for inhibiting immune-cell infiltration and promoting angiogenesis.

### 3.3 Multivariate cox regression based on bootstrap replicates uncovered the prognostic value of MGRRGs and the TME

**Figure 4:**
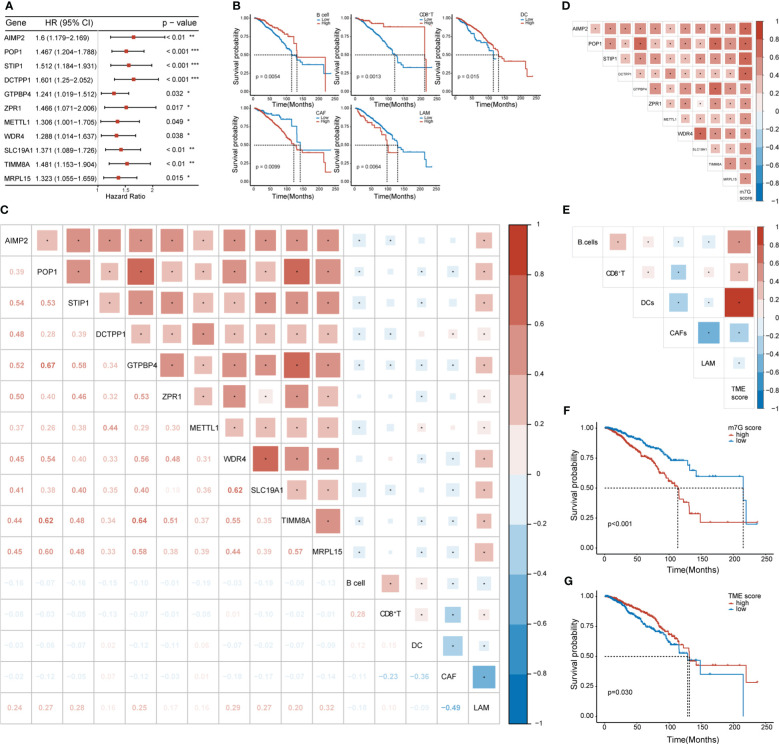
The prognostic values of MGRRGs and TME cells. **(A)** Forest plot of univariate Cox regression analysis of 11 prognosis-related MGRRGs. **(B)** Kaplan–Meier-curve analysis of OS for patients classified according to five prognosis-related TME cell types. **(C)** Correlation between TME cells and MGRRGs. **(D)** Pearson correlation analysis of prognosis-related MGRRGs and m7G scores. **(E)** Pearson correlation analysis of prognosis related TME cells and TME scores. **(F, G)** Kaplan–Meier-OS curves are shown for tumors with high m7G scores versus low m7G scores **(F)** and for tumors with high TME scores versus low TME scores **(G)**. CI, confidence interval; HR, hazard response. *p< 0.05; **p< 0.01; ***p< 0.001.

We conducted univariate Cox regression analysis on the expression matrix of MGRRGs and the cell-abundance matrix of 1,043 patients with BC in TCGA-BRCA dataset. We identified nine MGRRGs and five TME cells as prognostic factors (**Figures 4A, B**). Furthermore, correlation analyses of the MGRRGs and TME cells demonstrated a general negative correlation between MGRRGs and prognostically favorable TME cells, with a significant positive correlation with prognostically unfavorable lipid-associated macrophages (LAMs; **Figure 4C**). We then performed multivariate Cox regression analysis with 1000 bootstrap replicates to establish m7G and TME scores based on the prognostic characteristics of MGRRGs and the three prognostically favorable TME cells (coefficients shown in **Supplementary Tables 4**, **5**). Risk scores were calculated using the following formulas: m7G score = (0.55955 × *AIMP2*) + (2.22332 × *POP1*) + (0.75771 × *STIP1*) + (1.88989 × *DCTPP1* + (−1.40753 × *GTPBP4*) + (1.1109 × *ZPR1*) + (0.3425 × *METTL1*) + (−1.26914 × *WDR4*) + (1.28125 × *SLC19A1*) + (0.50205 × *TIMM8A*) + (−0.3904 × *MRPL15*); TME score = (−1.6721 × B cells) + (−1.73618 × CD8^+^ T cells) + (−1.53024 × dendritic cells [DCs]). Notably, our results demonstrated a strong positive correlation between m7G scores and prognostically unfavorable MGRRGs (**Figure 4D**). Moreover, we observed a positive correlation between TME scores and prognostically favorable TME cells and a negative correlation between the TME score and prognostically unfavorable TME cells (**Figure 4E**). Furthermore, patients with high m7G and low TME scores had significantly shorter survival times than those with low m7G and high TME scores (**Figures 4F, G**).

### 3.4 Prognostic value of the m7G-TME classifier

**Figure 5:**
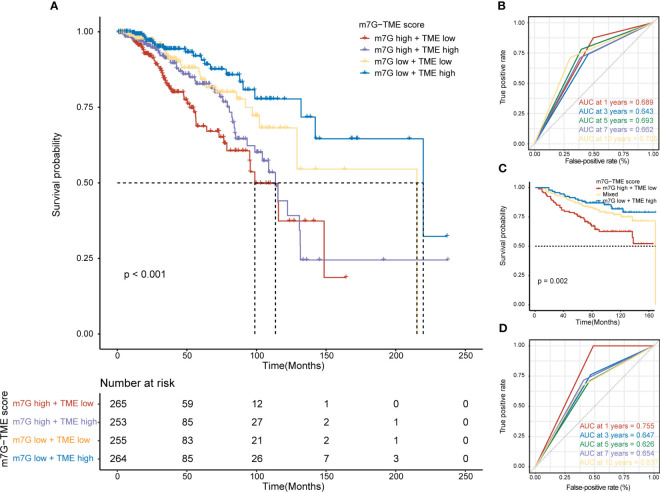
Prognostic value of the m7G-TME classifier. **(A)** Kaplan–Meier curves of the training cohort (*n* = 1043) are presented with patients categorized into four subgroups based on the m7G-TME classifier. **(B)** ROC curves for the 1-, 3-, 5-, 7-, and 10-year survival rates of the training cohort according to the m7G-TME classifier. **(C)** Kaplan–Meier curves for the testing cohort (*n* = 327) stratified by the m7G-TME classifier. **(D)** ROC curves for the 1-, 3-, 5-, 7-, 10-year survival rates of the testing cohort based on the m7G-TME classifier.

We found that MGRRGs correlated negatively with prognostically favorable TME cells and positively with prognostically unfavorable TME cells. Building on these discoveries, we created an m7G-TME classifier by merging the m7G score with the TME score, which enabled us to sort the patients into four subgroups, namely the m7G^low^+TME^high^, m7G^low^+TME^low^, m7G^high^+TME^high^, and m7G^high^+TME^low^ groups. In TCGA-BRCA dataset (*n* = 1043 patients), our m7G-TME classifier displayed a statistically distinct prognosis (**Figure 5A**) and could predict OS at 1, 3, 5, 7, and 10 years, with an area under the curve (AUC) range of 0.643–0.705 (**Figure 5B**). Our findings indicate that the m7G and TME scores significantly impact BC prognosis. Patients in the m7G^low^+TME^high^ subgroup had the most favorable prognosis compared with patients in the other three subgroups. The prognoses of patients in the m7G^low^+TME^low^ and m7G^high^+TME^high^ subgroups were less divergent, prompting us to consolidate these two subgroups into a mixed subgroup. Notably, we validated the performance of the m7G-TME classifier in another independent BC cohort, GSE20685 (*n* = 327; **Figure 5C**). The differences in prognoses were statistically significant in all three patient subgroups, with AUC values for the receiver operating characteristic (ROC) curves ranging from 0.626 to 0.755 (**Figure 5D**).

### 3.5 Tumor biological functions among different m7G-TME subgroups

**Figure 6:**
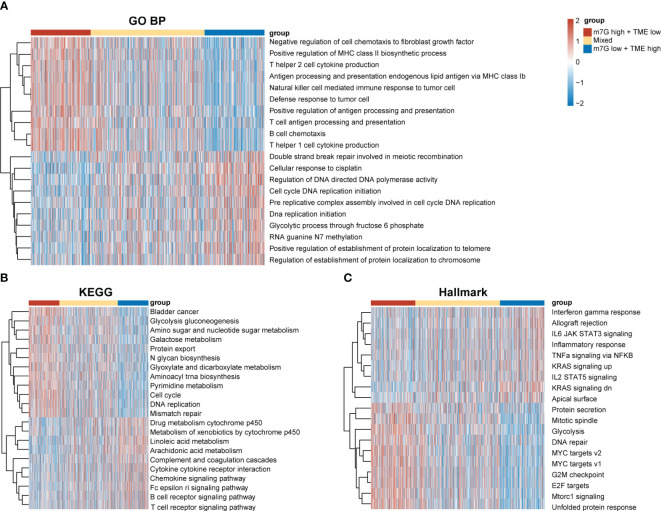
Differences in tumor biological functions and tumor immunophenotypes among different m7G-TME subgroups. **(A–C)** Heatmaps for GO-BP **(A)**, KEGG **(B)**, Hallmark **(C)** GSVA analysis.

Our results demonstrated significant prognostic differences in the m7G-TME classifier, which prompted us to perform tumor molecular-signature enrichment analysis based on gene-function annotations using the GO-BP (**Figure 6A**), KEGG (**Figure 6B**), and Hallmark gene sets (**Figure 6C**) for the m7G-TME subgroup. Interestingly, tumors in the m7G^low^+TME^high^ group displayed significantly higher enrichment for major histocompatibility complex (MHC)1-like molecule-mediated antigen-presentation responses, negative regulation of fibroblast chemokines, resistance in tumors, and multiple immune cell infiltration than those in the m7G^high^+TME^low^ group. Furthermore, tumors in the m7G^high^+TME^low^ subgroup showed much higher enrichment scores for terms related to cell-cycle progression, DNA replication, RNA translocation-related gene expression, and glycolytic pathways. Additionally, the results of the tumor molecular-signature enrichment analysis in the mixed group were intermediate between those in the aforementioned two groups. Our findings emphasize that patients in the m7G^low^+TME^high^ group had stronger antitumor immune responses and less tumor growth, whereas those in the m7G^high^+TME^low^ group had more aggressive tumors. These results underscore the importance of integrating m7G and TME scores to help understand prognostic differences between different subgroups based on tumor biology.

### 3.6 Association between the m7G-TME classifier and clinical features

**Figure 7:**
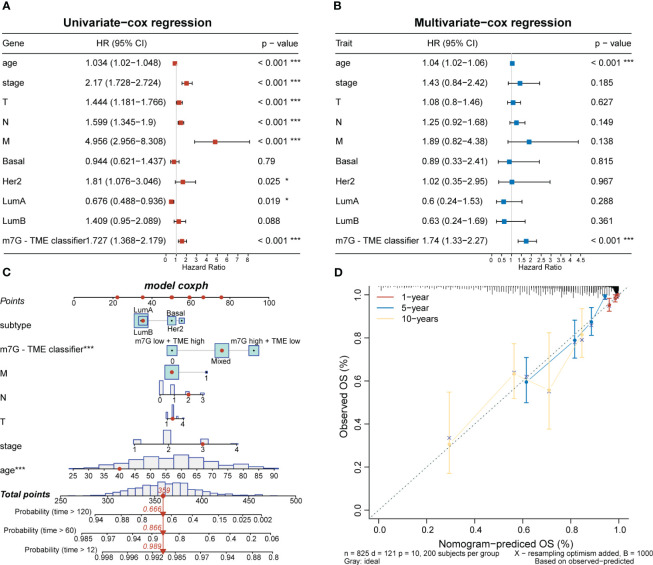
Associations with clinical features and the m7G-TME classifier. **(A, B)** Forest diagrams of univariate Cox regression analysis and multivariate Cox regression analysis of the m7G-TME classifier. **(C)** Nomogram of m7G-TME-classified subgroups and clinical characteristics for patient number 1. **(D)** Calibration plots were generated for the nomogram to evaluate the predicted probabilities. *p< 0.05; ***p< 0.001.

To investigate clinical characteristics based on the m7G-TME classifier, we combined the m7G-TME subgroups with clinical characteristics (including the age, tumor subtype, and tumor–node–metastasis stage). The results of our univariate and multivariate Cox regression analyses suggested that the m7G-TME classification is an independent prognostic risk factor (*p<* 0.001; **Figures 7A, B**). A nomogram was constructed for patient number 1 to facilitate analysis of the model (**Figure 7C**). Calibration curves were generated to evaluate the fit of the classifier model to real-world situations. The three fitted lines at 1, 5, and 10 years closely matched the reference line (**Figure 7D**), indicating that the prediction had high accuracy. Therefore, we validated the accuracy of the m7G-TME classifier for different clinical subgroups. Our classifier accurately assessed the prognostic risk of patients in various subgroups, including those with different ages, Luminal B and basal-like subtypes of BC, stage I–II BC and tumor stage 1–2, and the presence of lymph node metastasis (**Supplementary Figure 2**).

### 3.7 Immune phenotyping of the m7G-TME subgroups and predictions of immunotherapeutic responses

**Figure 8:**
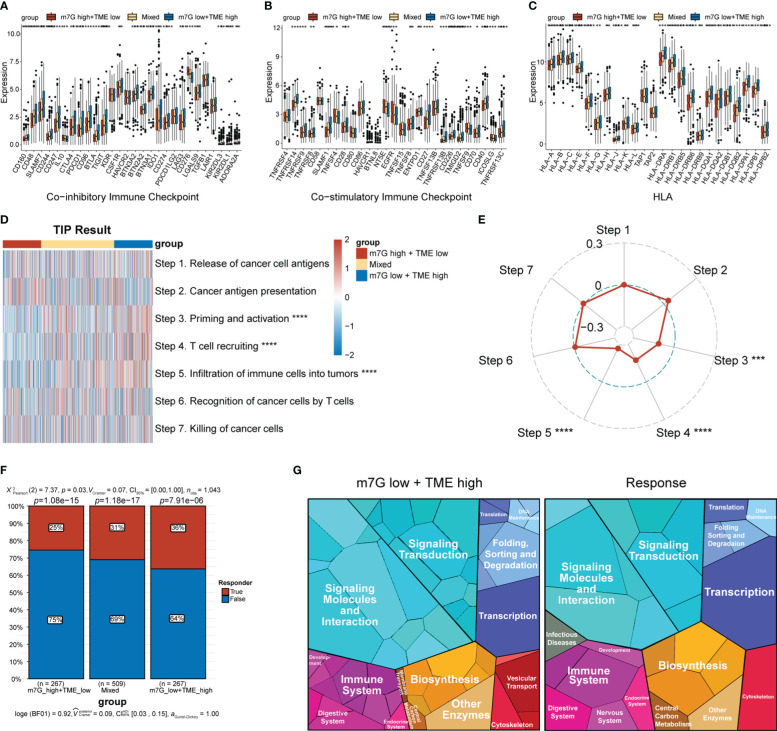
Predictions of ICI-treatment responses according to the m7G-TME classifier. **(A–C)** Box plots showing the expression level of MHC and immune-checkpoint markers in three subgroups, stratified by the m7G-TME classifier. **(D)** Heatmap for the results of TIP analysis. **(E)** The correlation coefficients between the m7G scores and specific steps in the tumor–immune cycle are illustrated in the radar plot. **(F)** TIDE-based predictions of the response rates of different subgroups based on the m7G-TME classifier. **(G)** Functional analysis in m7G^low^+TME^high^ group and TIDE-predicted responder group using proteomaps. Each small polygon in the figure corresponds to a single KEGG pathway, and its area indicates the abundance of proteins within that pathway. *p< 0.05; **p< 0.01; ***p< 0.001; ****p< 0.0001.

The expression levels of MHC and immune-checkpoint markers were assessed across different subgroups (**Figures 8A–C**). Interestingly, the m7G^low^+TME^high^ subgroup showed higher expression levels of MHC and most of the co-stimulatory and co-inhibitory receptors than the mixed and m7G^high^+TME^low^ subgroups. Differences in the cancer–immunity cycle among the various subgroups were also investigated. In the m7G^low^+TME^high^ subgroup, increased activity was observed at multiple steps in the cell cycle (**Figure 8D**), specifically at priming and activation (step 3), T cell recruiting to tumors (step 4), and immune cell infiltration into tumors (step 5). Correlation analysis between the m7G scores and steps in the tumor–immune cycle revealed significant negative correlations with steps 3–5 (**Figure 8E**). We assessed the capacity of the m7G-TME classifier to predict clinical responses to ICI therapies. The response rates to ICI therapies in different subgroups were predicted using the TIDE website, with patients in the m7G^low^+TME^high^ group exhibiting the highest response rate (36%) when compared to those in the mixed and m7G^high^+TME^low^ subgroups, with response rates of only 31% and 25%, respectively (**Figure 8F**). Moreover, proteomap visualization suggested how the m7G-TME classifier enabled therapeutic-response predictions for patients treated with ICIs (**Figure 8G**). Specifically, the proteomap revealed very similar patterns between the m7G^low^+TME^high^ group and the ICI-treatment responders. Overall, these findings indicate that the m7G-TME classifier may be useful in predicting patient responses to ICI treatments.

### 3.8 Validation of BC-specific m7G-regulators and co-expressed genes

**Figure 9:**
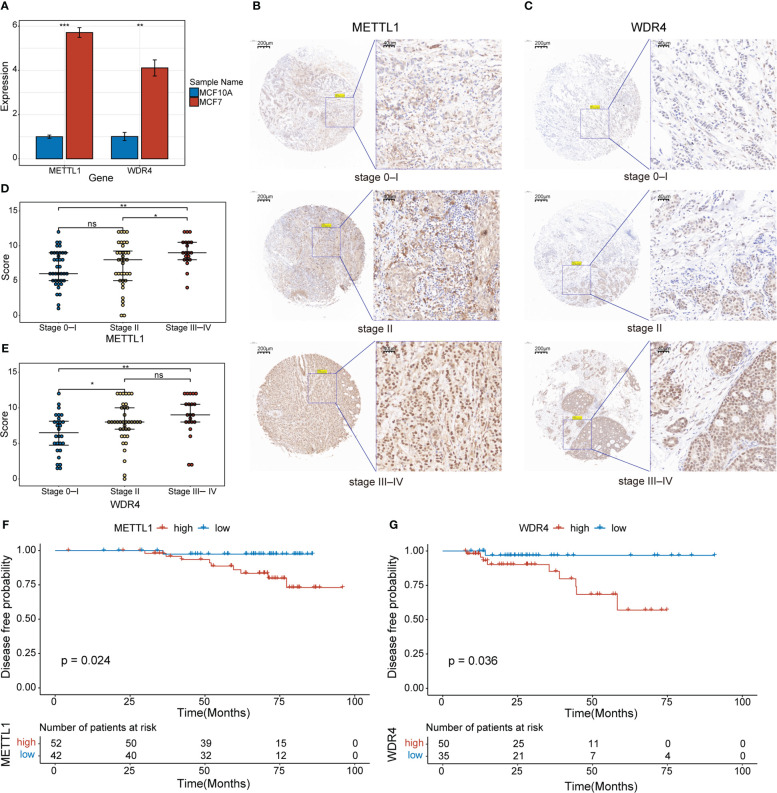
Clinical significance of METTL1 and WDR4 upregulation in BC cells. **(A)** RT-qPCR data showing the relative expression levels of *METTL1* and *WDR4* in MCF7 and MCF10A cells. **(B, C)** IHC staining for METTL1 and WDR4 in BC TMAs. Scale bars: left, 200 μm; right, 40 μm. **(D, E)** Comparison of METTL1- and WDR4-staining scores at different BC stages. **(F, G)** Kaplan–Meier curves displaying the differences in DFS rates between patients classified based on their IHC scores for METTL1 and WDR4 expression. ns, not significant *p< 0.05; **p< 0.01; ***p< 0.001.

Initially, we used qPCR to examine the expression levels of *METTL1* and *WDR4* in MCF-7 and MCF-10A cell lines, revealing that both genes were upregulated in the MCF-7 BC cell line (**Figure 9A**). To evaluate the clinical relevance of *METTL1* and *WDR4* in patients with BC, we performed IHC analysis of the METTL1 and WDR4 proteins with TMAs, quantifying the positive staining of tumor cells. As expected, METTL1 and WDR4 showed higher expression levels with increasing clinical stages (**Figures 9B–E**). Patients were divided into high- and low-expression groups based on the median score found with each microarray. DFS rates were calculated, followed by survival analysis using the Kaplan–Meier method and the log-rank test. The results suggested that patients with high *METTL1* and *WDR4* expression had shorter survival times than those with low expression of these genes (*p<* 0.05; **Figures 9F, G**). To ascertain the co-expression relationships between *METTL1, WDR4*, and other MGRRGs used to construct the model, MCF-7 cells were transfected with siRNAs targeting *METTL1* and *WDR4* mRNA. The qPCR results showed that downregulating *METTL1* and *WDR4* expression tended to decrease the expression levels of all nine MGRRGs (**Supplementary Figure 3**). Finally, we highlighted regions possibly involved in m7G modification in these nine MGRRGs by searching the m7GHub v2.0 database (**Supplementary Table 6**).

## 4 Discussion

In recent years, numerous studies on m7G modifications and the TME have provided a better understanding of their value in predicting the prognosis and treatment of patients with cancer. However, the association between m7G modifications and the TME in BC remains unclear. In this study, we conducted a combined analysis of m7G modification and TME signatures in BC to develop an m7G-TME classifier. Our findings revealed a promising predictive efficacy for estimating patient prognosis and immunotherapy responses.

METTL1 and WDR4 are the best described m7G regulators. METTL1 acts as an m7G methyltransferase, whereas WDR4 promotes the binding of heterodimeric complexes to target mRNAs, making it an m7G writer for mRNAs (39). Both *METTL1* and *WDR4* were expressed at high levels in BC, which correlated with a poor prognosis and underscored their significance in BC development. Over half of the *METTL1* and *WDR4* co-expressed genes were highly expressed in BC, and KEGG enrichment analysis revealed that these genes were significantly enriched for functions related to cell-cycle progression, DNA replication, and nucleoplasmic-transport pathways. Previous findings showed that deficiency of the METTL1–WDR4 complex in mouse embryonic stem cells led to significant dysregulation of the cell cycle and cell proliferation (40); therefore, we hypothesized that METTL1 and WDR4 may be associated with the cell cycle in BC cells. To explore the roles of *METTL1* and *WDR4* and co-expressed genes in the TME, we evaluated the expression levels of MGRRGs using scRNA-seq data. Intriguingly, MGRRGs were expressed at significantly higher levels in tumor cells than in non-tumor cells in the TME. Moreover, our GSEA results demonstrated that tumor cells with high MGRRG-expression levels exhibited a higher capacity for the epithelial–mesenchymal transition, which is associated with tumor metastasis and is involved in both malignancy and drug resistance in breast tumors (41). Our findings further confirmed the critical roles played by *METTL1* and *WDR4* and related genes in tumorigenesis.

Our findings indicate that tumor cells with high m7G scores exert significant autocrine and paracrine effects on the cellular communication network through multiple tumor growth-promoting signals (FGF5 and SEMA3C) and immunosuppressive signals (MIF and TGF-β). In BC, tumor cells promote the expression of *MEKK1* in CAFs by secreting FGF5. This phenomenon enhances the secretion of CCL5 in CAFs, which bind to CCR5 present on the tumor cells to induce tumor cell invasion (42). Meanwhile, SEMA3C can promote BC cell growth through autocrine secretion and mediate tamoxifen resistance in ER+ BC cells by activating MAPK and AKT signaling (43). MIF is overexpressed in nearly all types of solid tumors and is crucial in negatively affecting antitumor immunity, resulting in tumorigenesis and metastasis (44). Previous data demonstrated that MIF could evade antitumor immunity by activating myeloid-derived suppressor cells (MDSCs) and TAMs, working together to suppress cytotoxic T cell and natural killer (NK) cell activities (45). TGF-β, which is co-produced by tumor cells and stromal cells, activates signaling networks that influence TME formation (46). In addition to inhibiting NK and CD8^+^ cytotoxic T lymphocyte (CTL) functions, previous data suggest that TGF-β can promote the phenotypic transformation of M1-type macrophages to M2-type macrophages and enhance M2-type macrophage activity, assisting tumor metastasis and angiogenesis (47). The results of our study indicate that tumor cells with high m7G scores exhibit stronger communication with LAMs through MIF and TGF-β signaling communication. LAMs form a specific macrophage subset with a high expression of lipid-metabolism genes (such as those encoding fatty acid-binding protein 5 or apolipoprotein E). The results of recent studies have also shown that LAMs exist in the TMEs of BC and lung metastatic lesions (48) and contribute to an immunosuppressive microenvironment by promoting extracellular-matrix remodeling and inhibiting T cell activation and proliferation, leading to poor patient prognosis and immunotherapy resistance (49). Therefore, we postulated that tumor cells with high m7G scores can impede antitumor immunity by recruiting LAMs and enhancing their activity. Our results suggest a close association between m7G modifications and the TME.

Based on a review of existing literature, eight MGRRGs associated with BC prognosis that promote tumorigenesis in many other tumor types, have been identified (50–57). CD8^+^ T cells are key effector cells in antitumor immunity that collaborate with cytotoxic molecules, such as granzyme and perforin (58). Positive correlations between the abundance of CD8^+^ T cells and improved survival rates have been demonstrated in various cancers (59). In addition to their role in humoral immunity, B cells can act as antigen-presenting cells (APCs) and enhance cellular immunity. B cells have antitumor immune functions and may help improve patient prognosis. CD20^+^ B cells positively correlate with favorable prognoses in patients with non-small cell lung carcinoma and ovarian cancer, perhaps because they serve as APCs that amplify cytolytic T cell responses (60, 61). DCs are major APCs that present MHC class-I molecules to CD8^+^ T cells, secrete chemokines to recruit effector T and NK cells into the TME, and produce cytokines to sustain effector-cell cytotoxicity (62, 63). Our results suggest that infiltrating CD8^+^ T cells, B cells, and DCs in BC are associated with better prognosis. We observed negative correlations between multiple MGRRGs and these three immune-cell populations, as well as positive correlations with LAMs. The scRNA-seq data obtained in this study suggest a possible relationship between increased MGRRG-expression levels in tumor tissues and elevated LAM infiltration, which could inhibit the antitumor immune activities of DCs, CD8^+^ T, and B cells.

Our results revealed a strong association between m7G modifications and the TME. To prevent overfitting, we created the m7G and TME scores using bootstrap multivariate Cox regression and built the m7G-TME classifier to assess the prognosis of patients with BC. As expected, the m7G^low^+TME^high^ subgroup had the most favorable prognosis. In contrast, the m7G^high^+TME^high^ subgroup had a worse prognosis than the m7G^low^+TME^high^ subgroup and a similar prognosis to the m7G^low^+TME^low^ subgroup, potentially due to stronger m7G modifications that limited antitumor immune activities. The prognostic predictiveness of our classifier was confirmed across independent BC cohorts and various TCGA-BRCA clinical subgroups, indicating its broad applicability to patients with BC. The AUC values of the ROC curve demonstrated good accuracy in predicting the survival rates at distinct stages of BC development.

By elucidating the biological functions of these subgroups, we discovered that the m7G^high^+TME^low^ subgroup showed activation of several metabolic pathways, including glycolysis. Glucose metabolism in tumor cells permits the production of lactic acid even under oxygenated conditions, leading to decreased rates of oxidative phosphorylation. Abundant glucose consumption and lactic acid release ultimately result in an acidic TME. A low TME pH favors the selection of aggressive tumor cells, inhibits antitumor immunity, and facilitates tumor progression (64). A low TME pH also accelerates effector T cell exhaustion (65); increases the differentiation and accumulation of regulatory T cells, M2-like macrophages, and MDSCs (66); suppresses cancer immune surveillance; and promotes immune evasion. Our scRNA-seq data suggest that high MGRRG-expression levels corresponded to a reduced oxidative phosphorylation capacity, indicating a possible link between m7G modification and glucose metabolism. However, the precise regulatory mechanisms involved remain unknown and require further investigation. Collectively, our results underscore the importance of establishing an m7G-TME classifier and offer insights into the mechanisms underlying its prognostic and treatment-response-prediction capabilities.

We also observed high expression levels of multiple HLAs and immune checkpoints (co-inhibitory receptors such as programmed cell death 1 [PDCD1], CTL-associated protein 4 [CTLA4], CD274, and co-stimulatory receptors such as CD40) in the m7G^low^+TME^high^ subgroup. HLA plays a crucial role in antigen presentation during antitumor immunity, with high expression levels helping immune cells recognize and destroy tumors (67). Co-inhibitory receptors are immune checkpoints that maintain immune tolerance but are frequently co-opted by cancer cells to avoid immune surveillance. ICIs restore antitumor immune responses by interrupting co-inhibitory signaling pathways, primarily by targeting PDCD1, CTLA4, and CD274 (68). Co-stimulatory receptors are associated with activation of APCs, promotion of pro-inflammatory factors, and stimulation of anti-tumor responses in CD8+ T cells. Moreover, expression of the co-stimulatory receptor CD40 is associated with enhanced responsiveness of melanoma to immune checkpoint blockade therapy (69). Furthermore, we observed significant differences in circulating anti-cancer immune cells across the subgroups, with the m7G^high^+TME^low^ subgroup exhibiting weaker capacities in terms of antitumor-immunity initiation and activation, T cell recruitment to the TME, and immune cell infiltration into the TME. The negative correlation between the m7G score and step 5 of the cancer–immunity cycle further suggests that high levels of m7G-modified BC cells may inhibit immune-cell infiltration into the TME. These results confirm our scRNA-seq findings, emphasizing the potential of the m7G-TME classifier for the pre-immunotherapy stratification of patients with BC. The TIDE platform could be used to assess the power of the m7G-TME classifier to predict immunotherapeutic responses and indicated that the m7G^low^+TME^high^ subgroup had the highest immunotherapy-response rate in TCGA-BRCA dataset. The similarity in the proteomic patterns between the m7G^low^+TME^high^ subgroup and the immunotherapy-response group may shed mechanistic insight as the why the former group was well suited for ICI treatments, further underscoring the therapeutic predictive value of the m7G-TME classifier.

This study has certain limitations. First, the gene expression-based m7G-TME signature requires further validation using immunofluorescence or flow-cytometric analysis of tumor samples (biopsies). Second, owing to the absence of a large ICI-treatment cohort for BC, the ability of the m7G-TME classifier to predict immunotherapy responses requires further validation.

In conclusion, integrating m7G modifications and cellular-landscape signatures within the TME facilitated the prediction of prognosis and treatment responses. Our findings offer a viable approach for prognostic estimation and patient stratification for managing BC in the future.

## Data availability statement

The datasets presented in this study can be found in online repositories. The names of the repository/repositories and accession number(s) can be found in the article/**Supplementary Material**.

## Ethics statement

The studies involving humans were approved by Medical Ethics Committee of Quanzhou First Hospital of Fujian Medical University. The studies were conducted in accordance with the local legislation and institutional requirements. The participants provided their written informed consent to participate in this study.

## Author contributions

TL: Conceptualization, Investigation, Software, Formal Analysis, Visualization, Writing - original draft. ZC: Resources, Validation, Writing - Review & Editing. ZW: Data curation, Investigation, Writing - original draft. JL: Data curation, Investigation. DC: Supervision, Project management, Methodology, Funding acquisition, Writing - Review & Editing. 
