# Supplementary material for: Combined signature of N7-methylguanosine regulators with their related genes and the tumor microenvironment: a prognostic and therapeutic biomarker for breast cancer
Source: Front Immunol. 2023 Oct 5;14:1260195. doi: 10.3389/fimmu.2023.1260195 (PMC10585266; doi:10.3389/fimmu.2023.1260195)
Supplement: Supplementary file 1 [file DataSheet_1.docx]

Supplementary Material

# Supplementary Figures and Tables

## Supplementary Figures

**
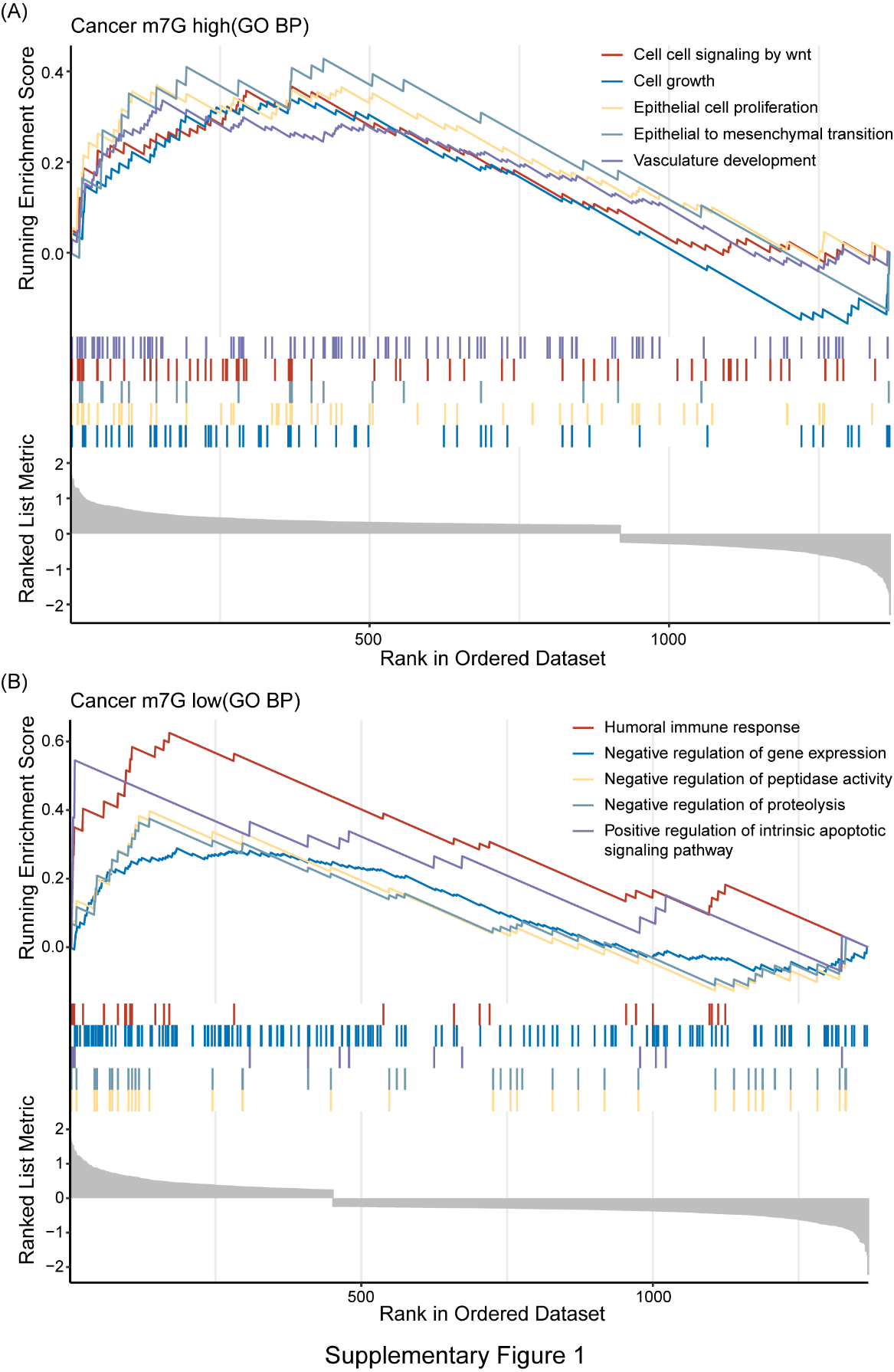
**

**Supplementary Figure 1.** GSEA results for the GO-BP gene set of cancer cells with high (A) and low (B) m7G scores.


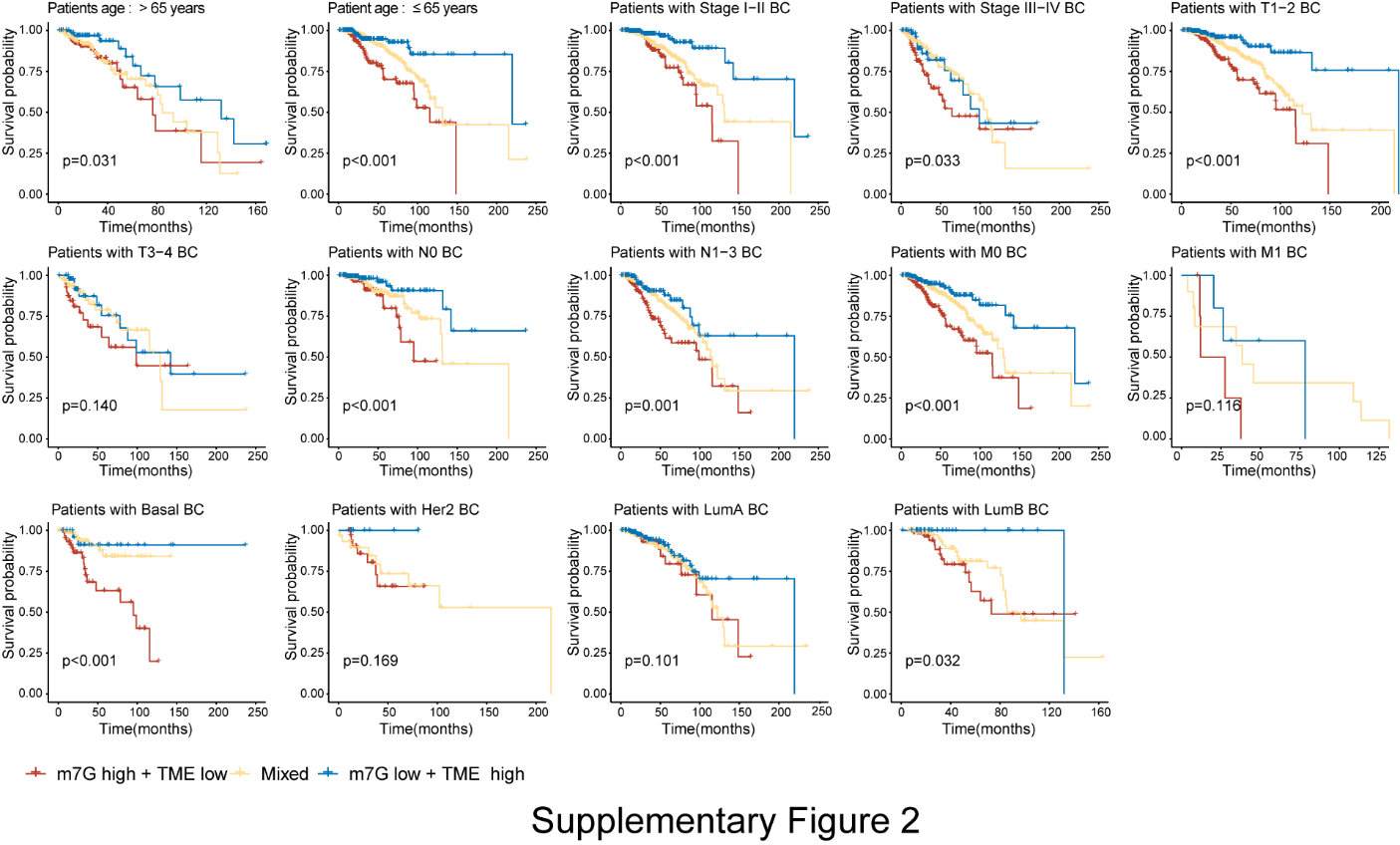


**Supplementary Figure 2.** Kaplan–Meier curves obtained using the m7G-TME classifier with clinical subgroups.


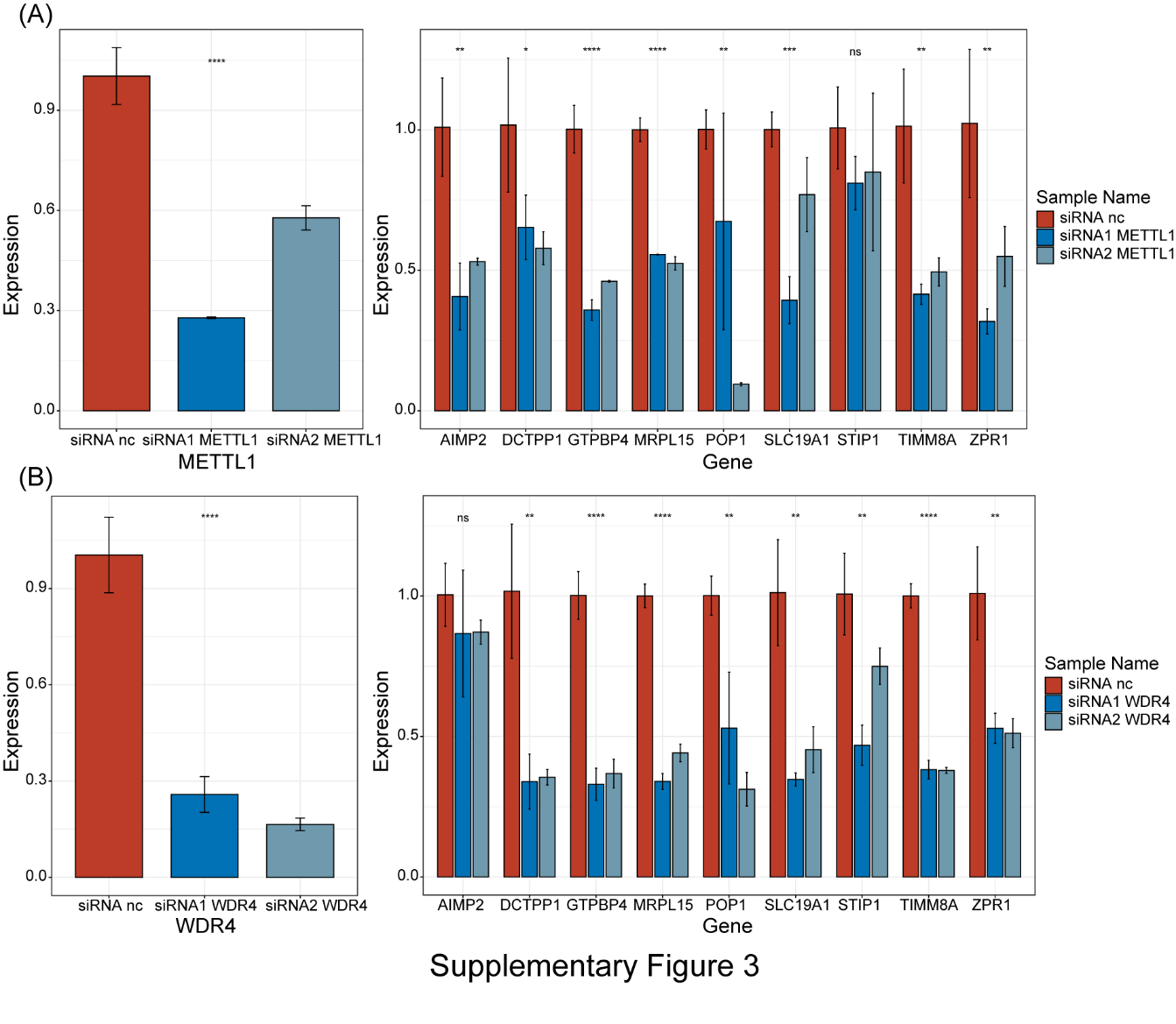


**Supplementary Figure 3.** Expression of MGRRGs between groups with METTL1 (A) or WDR4 (B) expression and the control group. **p* < 0.05; ***p* < 0.01; ****p* < 0.001; *****p* < 0.0001.

## Supplementary Tables

**Supplementary Table 1.** Sequences for primers used in this study

| **Gene** | **Primer_F(5'–3')** | **Primer_R(5'–3')** |
| --- | --- | --- |
| METTL1 | GAGCCACGATGACCCAAAGGATAAG | CAGCGGTGACAGTTCCACTAACAG |
| WDR4 | ATAGCCTCGGAGGAGAAGGTCTTG | CAGGACTCACAGCCACATCTAACAG |
| AIMP2 | CCGCTCCATGAACTCTGCTCTTG | ATTGGCTGGCACTGTCACACTG |
| POP1 | GCCACTTTCCCACTCCATCCTAAC | GGTGTCTCCTCTGTGTCCTCTCC |
| STIP1 | ACCAATCCGTGTCGCAGAAGTTC | GGAGTAGCACTGTAAGGCATCATCG |
| DCTPP1 | ACCCATTCTCGTGTTTCCCTGTTG | CACCATCTGGACTCTTACAGCCTTC |
| GTPBP4 | TGCCAACCATTGATCCGAATACCAG | CGTCTGCTCTCGTCACCTTGTTG |
| ZPR1 | TGGGTGGGTGGAAGTTCAGAGG | TGGTGTCAGCAGGCATTCAGAAAG |
| SLC19A1 | CTCAGCTTCGTGTCGGTGT | AGCGAGATGTAGTTGAGCGTG |
| TIMM8A | AAGGGTGACTCCGCTTCTCTGG | GTGCCTGAAAGTGCTGGGTGTC |
| MRPL15 | GGCTCCAAGAAACCGGAGAG | GCGTCTGAAACTATGTCCTTCGT |

**Supplementary Table 2.** Sequences of METTL1 and WDR4 siRNAs

| **siRNA** | **Sense (5'–3')** | **Antisense (5'–3')** |
| --- | --- | --- |
| siRNA1 METTL1 | CUUCCUAACUUCUUCUACA | UGUAGAAGAAGUUAGGAAG |
| siRNA2 METTL1 | CACACUUAUUCUGGGUCUG | CAGACCCAGAAUAAGUGUG |
| siRNA1 WDR4 | CCCAUAGCAUCGAGUCCUU | AAGGACUCGAUGCUAUGGG |
| siRNA2 WDR4 | GACGAGAAGAUCCGAGUCA | UGACUCGGAUCUUCUCGUC |

**Supplementary Table 3.** Correlation coefficients for MGRRGs and m7G regulators and database-validation results (see Supplementary Table 3.xlsx for details)

**Supplementary Table 4.** Coefficients of prognosis-related MGRRGs

| **MGRRG** | **Coef** | **boot_SD** | **boot_coef** |
| --- | --- | --- | --- |
| AIMP2 | 0.122 | 0.218 | 0.560 |
| POP1 | 0.413 | 0.186 | 2.223 |
| STIP1 | 0.143 | 0.188 | 0.758 |
| DCTPP1 | 0.283 | 0.150 | 1.890 |
| GTPBP4 | -0.304 | 0.216 | -1.408 |
| ZPR1 | 0.247 | 0.222 | 1.111 |
| METTL1 | 0.064 | 0.186 | 0.342 |
| WDR4 | -0.271 | 0.214 | -1.269 |
| SLC19A1 | 0.236 | 0.184 | 1.281 |
| TIMM8A | 0.101 | 0.202 | 0.502 |
| MRPL15 | -0.077 | 0.197 | -0.390 |

**Supplementary Table 5.** Coefficients of prognosis-related TME cells

| **TME cell** | **Coef** | **boot_SD** | **boot_coef** |
| --- | --- | --- | --- |
| B cells | -4.471 | 2.674 | -1.672 |
| CD8^+^T cells | -35.144 | 20.242 | -1.736 |
| DCs | -1.529 | 0.999 | -1.530 |

**Supplementary Table 6.** m7G region of MGRRGs

| **MGRRG** | **Position** | **Region** |
| --- | --- | --- |
| AIMP2 | 6012935-6013009 | AAATAAGAGCAGCAGACTAGGAGAGGTGAGAAAGTGCTGATTAAGCCCGAAGGTAAATAGGAGTTAAGCAAACAA |
|  | 6009259-6009288 | CCGCAGCAGGGTCAGAAGGGAGGTGGCCGG |
|  | 6009258-6009288 | CCCGCAGCAGGGTCAGAAGGGAGGTGGCCGG |
|  | 6023353-6023384 | GGCGAAGGGAACATTGCACGTTTCTTGTTCTC |
|  | 6009329-6009360 | TGGCACGCGCTACCCCCTTTTGCTTTGGTTCT |
|  | 6015211-6015224 | TGAGTTGAAAGCTG |
|  | 6015289-6015352 | CCAAGCGGATGAGCCCACGACTTTAACCACCAATGCGCTGGACTTGAATTCAGTGCTTGGGAAG |
|  | 6017814-6017924 | GATTACGGGGCGCTGAAAGACATCGTGATCAACGCAAACCCGGCCTCCCCTCCCCTCTCCCTGCTTGTGCTGCACAGGCTGCTCTGTGAGCACTTCAGGGTCCTGTCCACG |
| POP1 | 98128394-98128425 | GAAATCAGTGCTATGTTAAAAGCTGTGACCCA |
|  | 98133945-98133976 | GCAGGATTTATCCTATTACTGTTGTTTGGAGT |
|  | 98136520-98136669 | GGAACCCATCAAATCAGCTGTCTGCATCGCTGACCCACTTCCAACACCATCCCAAGAAAAAAGCCAAACTGAATTGCCTGACGAGAAAATTGGCAAGAAAAGAAAAAGGAAAGATGATGGAGAAAATGCTAAACCAATTAAAAAAATTAT |
|  | 98136570-98136738 | CCCAAGAAAAAAGCCAAACTGAATTGCCTGACGAGAAAATTGGCAAGAAAAGAAAAAGGAAAGATGATGGAGAAAATGCTAAACCAATTAAAAAAATTATCGGTGATGGAACTAGAGATCCATGTCTACCATACTCTTGGATCTCTCCAACCACAGGCATTATAATCAG |
|  | 98136597-98136628 | CTGACGAGAAAATTGGCAAGAAAAGAAAAAGG |
|  | 98156262-98156293 | ATGAAGTGGGCACATCCATAGAGCACCCCAGG |
|  | 98156306-98156337 | GTAATGGATGCAGGGTGTCAAGAATCGGCAGG |
|  | 98159554-98159678 | TTTCTAAAAAATGTAAATATGCAGTTAGGCATTATTTTATGTAAATGCATTGGGTTTTTACTGTAGCATTTGGCACTAAATGGCTTTGGGGGTGATGAGGTGGGGAAGGATACAGCAGGTGGTAC |
|  | 98159729-98159828 | TACCATGCCTCTGAATGTCTTTGGATCCAACCCAGATGAGACTGAAAAAAAAAAAACAGTGTAACTAAGTGGCATCTGTAAACAGAATAAATGAAAATGT |
| STIP1 | 64185922-64185974 | CAATCCGTGTCGCAGAAGTTCGCTCCTCCCTCCATTCGTGGAGCCTGAGATGG |
|  | 64186171-64186217 | AGCTTCTAGTAGGTTCCAGAAGGCGGCGCGTGCGGTTGGGAACGCGG |
|  | 64193088-64193119 | TGAAGGAGAAAGGCAACAAGGCCCTGAGCGTG |
|  | 64194180-64194211 | TGGTTTAAGGGCTATTCACGAAAAGCAGCAGC |
|  | 64195702-64195876 | GGGCAGTATGGATGAGGAGGAAGAGATTGCAACACCTCCACCACCACCCCCTCCCAAAAAGGAGACCAAGCCAGAGCCAATGGAAGAAGATCTTCCAGAGAATAAGAAGCAGGTCTTGTTTTTTTCTCTCCTCACTGTCACCTATCTATAAACAACTAGAAATCTTTATGTTGAA |
|  | 64195715-64195720 | GAGGAG |
|  | 64195715-64195723 | GAGGAGGAA |
|  | 64195727-64195876 | ATTGCAACACCTCCACCACCACCCCCTCCCAAAAAGGAGACCAAGCCAGAGCCAATGGAAGAAGATCTTCCAGAGAATAAGAAGCAGGTCTTGTTTTTTTCTCTCCTCACTGTCACCTATCTATAAACAACTAGAAATCTTTATGTTGAA |
|  | 64196836-64196860 | ACTCAGCCGCTTCTCATCCTCTCAC |
|  | 64196936-64197110 | CTCAGAATAAAGCCTGGTCCAAAACTCTGCTGTCCTCACCGGTTGCTTCAAGTCGAAGGGATTTCCTCTCCCCTAAAGAAGCGCTCTGCTTCCTGACAAAGATCTAGGAGAGTAGGTGGAAAAGGGGGAGTTTCAGAGCAAGCAAAGATGAAGTTAGTTTTCATTCTAACGGCTG |
|  | 64197161-64197176 | ATAGAATTCTATTCAT |
|  | 64197190-64197223 | CAGAACTTGTGACAGATCATAGATTCTTGCTTTG |
|  | 64197199-64197397 | TGACAGATCATAGATTCTTGCTTTGTCACCTGAGTTAGATTTGCTCAGCACTCACTTCTAAACCTCATCTAGGCACTGAAAGAAAAAGAGCTGGGGAACGATGCCTACAAGAAGAAAGACTTTGACACAGCCTTGAAGCATTACGACAAAGCCAAGGAGCTGGACCCCACTAACATGACTTACATTACCAATCAAGCAG |
|  | 64197308-64197321 | AGAAGAAAGACTTT |
|  | 64197493-64197568 | CGGTATACTTTGAAAAGGGCGACTACAATAAGTGCCGGGAGCTTTGTGAGAAGGCCATTGAAGTGGGGAGAGAAAA |
|  | 64197502-64197533 | TTGAAAAGGGCGACTACAATAAGTGCCGGGAG |
|  | 64197544-64197593 | AGGCCATTGAAGTGGGGAGAGAAAACCGAGAAGACTATCGACAGATTGCC |
|  | 64197546-64197564 | GCCATTGAAGTGGGGAGAG |
|  | 64197842-64197871 | TTTCTTTATCAGAGCATATGCTCGAATTGG |
|  | 64199967-64200036 | CGGCTGGCCTACATAAACCCCGACCTGGCTTTGGAGGAGAAGAACAAAGGCAACGAGTGTTTTCAGAAAG |
|  | 64200179-64200186 | CCAGGCCA |
|  | 64200183-64200210 | GCCATGAAGCATTATACAGAAGCCATCA |
|  | 64200183-64200212 | GCCATGAAGCATTATACAGAAGCCATCAAA |
|  | 64203541-64203572 | CTGAGGTGCAGCAGATCATGAGTGACCCAGCC |
|  | 64203868-64203992 | GGCTCAAGAGTAGGACTGGCAAGTTCTGCGAAGTGGAGCAGGCCTCTGCAGCTCGGCGCCCCGGGCCTCGCCAGGACCCCTCCCTGCCGGGGCCTTCTGTAGAGGGGGTTGAATTGGGGCTTGCT |
|  | 64204142-64204173 | CCCTTCCCTTCGCCCTCATGTGGAAAGAGGAG |
| DCTPP1 | 30423665-30423739 | AGCTTCCTTGTCTATCAAATAAAAAGAATAGTACCTGCCCAATAGGGTCGTGAGGTTTCAATGAGTTATGTGAAT |
|  | 30423665-30423764 | ACTCAAATTAATCGTTCTGCACTTCAGCTTCCTTGTCTATCAAATAAAAAGAATAGTACCTGCCCAATAGGGTCGTGAGGTTTCAATGAGTTATGTGAAT |
|  | 30423745-30423771 | TGACATTACTCAAATTAATCGTTCTGC |
|  | 30423879-30423902 | TTTCTAGCAAATTCCCAGTAGGAT |
|  | 30424453-30424484 | GAACGGGCAGCCCTTCAAGAGGAGCTTAGTGA |
|  | 30424517-30424547 | TGTTCTGCCCCTAGTCAGTGGAAAACCGATG |
|  | 30429100-30429130 | TGGGAACAGTTCCATCAGCCTCGGAATCTCC |
|  | 30429130-30429161 | CCTCCATGCTGAGTTTGCTGCGGAACGAGACT |
|  | 30429238-30429289 | GAATTAGTTGCAGAATCTCCTGGTCCCAGGCCCCATTACATACTACGGGGGT |
|  | 30429589-30429686 | TTGGGTGGTGCCTCCGAGTGGTAACTAGGAACGCCCTTCAGCCTAGCCCTGGGAAGATACCGGCACCAGATACCGCCTCTGCTCTCCAGGCGCCGGGG |
|  | 30429919-30429923 | CCCGG |
|  | 30429919-30429929 | GCTGCTCCCGG |
|  | 30429995-30430026 | GTGGCTCGTGGGTTTTCCGTGAAGTCGCGGTG |
| GTPBP4 | 1000998-1001013 | TTCTGAAGATGATCAG |
|  | 1005818-1005907 | AAAATATTTACAGATTTGCAGTCTGAAGGATTCCCTGTAATAGAGACCAGCACCCTGACTGAGGAAGGTGTTATTAAAGTTAAAACAGAG |
|  | 1007018-1007061 | GCTTGCGATAGGCTTTTGGCTCATCGAGTGGAAACCAAAATGAA |
|  | 1007037-1007136 | CTCATCGAGTGGAAACCAAAATGAAGGGAAATAAAGTGAATGAGGTGCTGAATAGACTGCACCTGGCTATCCCAACCAGGAGGGACGATAAGGTAAGACG |
|  | 1007262-1007320 | TTCTTGCTTACATAAAGGATCGAGAAGGTGAAGAAAGTGCAGAACGCCACATGGCACCC |
|  | 1008958-1008998 | GAGAGGCCCCCTTTCATCCCTGAAGGAGTGGTGGCTCGCAG |
|  | 1008958-1009035 | GAGAGGCCCCCTTTCATCCCTGAAGGAGTGGTGGCTCGCAGGAAGAGGATGGAAACTGAGGAGTCCAGGAAGAAGAGG |
|  | 1009529-1009580 | GAACGAGATCTTGAGCTGGAAATGGGAGATGATTATATTTTGGATCTTCAGA |
|  | 1010420-1010520 | AGTACTGGGATTTAATGAATTTGTCTGAAAAACATGATAAGATACCAGAAATCTGGGAAGGCCATAATATAGCTGATTATATTGATCCAGCCATCATGAAG |
|  | 1012400-1012434 | CACACTCACTGACTCAGGGGTTTTCTCATTGTTAA |
|  | 1012400-1012662 | CACACTCACTGACTCAGGGGTTTTCTCATTGTTAATTTTGCTTCATTACAACTCCATTTTTAAAGAAATTGGAAGAATTAGAAAAAGAAGAAGAGCTGAGAACAGCTGCTGGAGAGTATGACAGTGTATCTGAGAGTGAAGACGAAGAGATGCTGGAAATCCGACAGCTGGCAAAGCAAATTCGAGAGAAAAAGAAGTTGAAAATTCTGGAGTCCAAAGAAAAGAATACACAGGGACCCAGGATGCCGCGAACTGCTAAGAAG |
|  | 1012553-1012584 | TGGAAATCCGACAGCTGGCAAAGCAAATTCGA |
|  | 1014247-1014268 | GTTCAGAGGACAGTTTTGGAGA |
|  | 1015859-1015896 | GAACTCCACGTGACGTTTCTGGTCTTAGGGATGTCAAG |
|  | 1017075-1017211 | ATGGTGAAGAAAGCCAAGACTATGATGAAGAATGCTCAGAAGAAGATGAATCGGTTGGGGAAGAAAGGGGAGGCGGATAGACACGTGTTTGATATGAAGCCCAAGCACTTGCTGTCTGGGAAGAGGAAAGCTGGTAA |
|  | 1017202-1017233 | AAGCTGGTAAAAAGGACAGGAGATAGTATCCG |
|  | 1017587-1017661 | CGTGCTTAAAATACGCTCTTAAATTATTTTCTAGTCTTATTTTACAATGTCTCATTGTAGTCTGTCTTCAACTAT |
|  | 1019487-1019686 | TACACATGGCTGACTCGACCTCCCTGCCTCTCACACTCTGTGTATTTTGTGAAGCTCCACAAACGGGGTCACGTCATCCAGGTGAGGCCACCACCGGTACAGAAACCTCTCGGCAATGGTTCTTAGCCAGGGGGTGACTTTGACTTCACCCCTCGCCTCCCTCTCCAGCAGCTCCCACAGCTCCTCCTGGGACAGGTAGA |
|  | 992487-992518 | AGGACTTCATAGACCTCACGTTGTCGAAGACT |
| ZPR1 | 116774974-116775148 | AACCCATGCCAGTCAGACTCTGAAACCTGAGGTCTGAATATTTTAAGCTCAGCTCCACAATTACTGTATTTGTTCTATTAGATACAACTGCAGAGAGAGGCCAAAGCTCAGAGCAGGCCATTGGCACGGCATCTGATCCTGCTTAACTGCCCTGCAGCACAGGGTGGAGACACTT |
|  | 116776824-116777148 | CACTTTGCCTCCTCCCCTCTTACTAAATTTGTTTCTGAGAGTTCTGCTTTCAGACATCTCTCCATATCAGATTCTTTGGCATCTTACTATCATGTGGGACTAACTTCCATACCTGTATCTCTAATTTCTGTAAGTGAGGGCGAGAGTAAGGTGTTTGGAGACTCAGGTTAGCAAATGACTCATTCAAACTATAAAGCTTCAGTACTTTGGGCAAACTTCTCACAGGCAACTATTCTACCCTAAGAGATTATCTCTAGGTATGTTTCCAGCTGACTTGGGAATCTAAGTTTACCAGACAACCCTCTGATCTATCATTTGGTCTCTT |
|  | 116777724-116777798 | TGCCTCTCATAAACAAAAGGTGACTCCTGTGGTGCAGACTGCAGGGTATTTGTTCAATAAAGACCAGTTCATTGA |
|  | 116778049-116778298 | TATCTACTTTATGCCAAGCACACCACAGAACAGAGGACAAAATAAATCTCTTTCATGGAACTTGAAGTCTAGTGGGGAAAAATGACAATAAACAGATCAGGAAAACAGTACATCAGATGGTGATTAGTGTCATGGAGGAATGTAAAGCAGGGAATGGGGCTGGGTTGTGCAAGGAAATGGGGAGTAGGGAAGATCTTCTGAACAAGAAGGTGATAATTTGACATTTGGGCAAAGCTGAGGAGGAGGTCAG |
|  | 116785861-116785892 | TTCATGGCAGGCAAACAAAGATGCTACAGCTG |
|  | 116787989-116788020 | AAGCCGAGGTCTGAATTGCGCGTGGTGGCCAT |
| SLC19A1 | 45562955-45563029 | AAGGGTCACTTCTGCTTCTCTTTCTGATGGTCTTTCCCTGGCCTTGAGTATTTGCCTCTCACACCTGCACAGATC |
|  | 45525890-45525921 | GCCCTGGTCTTCGGGGTCAACACGTTCTTTGC |
|  | 45537991-45538014 | TTCCAGGCACAGCGTCACCTTCGT |
| TIMM8A | 101346386-101346435 | ATTGAAGAAACAGCTATGAGAGGATTGGCTCCCATCTTTTGTTACTCTTG |
|  | 101348136-101348310 | GGTATCAAAGAAGACTTATGCCACTGGACACCCAGCACTTTCAGGCACCCGAGGTTGAAGCTGCCTAGTGCTGGTCACCAAGACTGATCGGAGCCCCCAGTACTGGGATTTTATTTTTGGCAGCCCGAGTCCCAGAGAGTCAGTGAATCAGTGAATCTGCTATTCCTGGACTCCT |
|  | 101348436-101348610 | TTGCAGCATTTCATCGAGGTAGAGACTCAAAAGCAGCGCTTCCAGCAGCTGGTGCACCAGATGACTGAACTTTGTTGGGTGAGGAGCCTGGGGCTGGGACCTGAACACTGTACCTACTTTCCCTCTGTCCTTGTACCGCGGGACTCGCGACAACGTCGGGGATTCGGGGTGGGGG |
| MRPL15 | 54135316-54135391 | GGCCCTGGACCTACTCCGGGGCCTGCCGCGTGTGAGCCTGGCCAACTTAAAGCCGAATCCCGGCTCCAAGAAACCG |
|  | 54135366-54135391 | AGCCGAATCCCGGCTCCAAGAAACCG |
|  | 54136508-54136531 | CAGGAGAGAAGACCAAGAGGTCGG |
|  | 54136508-54136556 | CAGGAGAGAAGACCAAGAGGTCGGAGAAGAGGTAGAAAATGTGGCAGAG |
|  | 54136508-54136606 | CAGGAGAGAAGACCAAGAGGTCGGAGAAGAGGTAGAAAATGTGGCAGAGGCCATAAAGGAGAAAGGCAAAGAGGAACCCGGCCCCGCTTGGGCTTTGAG |
